# The impact of reducing the femoral stem length in total hip arthroplasty during gait

**DOI:** 10.1007/s00402-021-03852-w

**Published:** 2021-03-24

**Authors:** Anatole Vilhelm Wiik, Adeel Aqil, Bilal Al-Obaidi, Mads Brevadt, Justin Peter Cobb

**Affiliations:** MSK Lab, Imperial College London, Michael Uren Hub, 2nd Floor, White City Campus, Wood Lane, London, W12 0BZ UK

**Keywords:** Gait, Biomechanics, Short stem total hip arthroplasty

## Abstract

**Aim:**

The length of the femoral stem in total hip arthroplasty (THA) is a practical consideration to prevent gait impairment. The aim of this study was to determine if reducing the femoral stem length in THA would lead to impaired gait biomechanics.

**Methods:**

Patients uniformly with the same brand implant of differing lengths (100 mm vs 140–166 mm) were taken retrospectively from a prospective trial introducing a new short stem. Twelve patients without any other disorder to alter gait besides contralateral differing length stem THA were tested at differing gradients and speed on a validated instrumented treadmill measuring ground reaction forces. An anthropometrically similar group of healthy controls were analysed to compare.

**Results:**

With the same posterior surgical approach, the offset and length of both hips were reconstructed within 5 mm of each other with an identical mean head size of 36 mm. The short stem was the last procedure for all the hips with gait analysis occurring at a mean of 31 and 79 months postoperatively for the short and long stem THA, respectively. Gait analysis between limbs of both stem lengths demonstrated no statistical difference during any walking condition. In the 90 gait assessments with three loading variables, the short stem was the favoured side 51% of the time compared 49% for the long stem.

**Conclusion:**

By testing a range of practical walking activities, no lower limb loading differences can be observed by reducing the femoral stem length. A shorter stem demonstrates equivalence in preference during gait when compared to a reputable conventional stem in total hip arthroplasty.

## Introduction

Bone and soft tissue preservation during arthroplasty continue to be at the forefront of twenty-first century research (1). Presently there is great interest in short stems for total hip arthroplasty (THA) (2–5). The desire to optimise femoral bone stock, morphology, load transfer, and stress shielding are the drivers for these feelings (6). In addition, an increasing number of younger patients require surgery who frequently have narrower diaphyseal canals, which make conventional longer stems more of a surgical challenge. In this challenging group, a shorter stem may provide an easier and potentially bone conserving alternative. Recent systematic reviews and meta-analysis have found comparable early to mid-term results for radiographic assessment, implant survivorship and patient reported outcome measures, while biomechanical studies have found more physiological stress shielding in a shorter stem (7–9). On the contrary, short stems are at risk of insertional malalignment and have been found to have a lower primary load at failure when compared to a conventional stem (2, 10, 11). Yet, hip function, arguably the most important outcome for patients, following the implantation of these devices remains comparatively unaddressed and benefits of either may be of interest (5, 12). A retrospective gait study attempted to shed light on this subject, however, conclusions were limited by its cohort design, which was open to selection bias (12). Gait analysis of subjects with both a short and long-stemmed THA on opposing sides provide a better control for patient associated variables, allowing a more objective measure of any functional differences conferred by the use of stems of different length. This type of study design has been successfully used before to demonstrate substantial functional differences between resurfacing and conventional total hip replacement implants (13). Current gait methods suggest that between limb differences are most pronounced when walking assessment occurs under challenging conditions (13). Thus, the aim was to determine if any hip impairment existed by reducing the stem length by comparing the ground reaction forces (GRFs) of the patient’s limb that were implanted with one long and one short stem hip replacement whilst walking up to their top walking speed and incline. Furthermore, as differing patients are likely to perform at a variety of speeds and levels, the second aim was to complete a comprehensive analysis of all speeds and inclines to determine if there is a favoured side during gait. Lastly, and in order to put the findings into perspective a healthy control group was measured as a benchmark. The null hypothesis was therefore limbs with a shorter stemmed THA would have a gait that is not distinguishable from limbs with a longer stem.

## Material and methods

### Participants

All arthroplasty subjects were identified from a registered multicentre and ongoing Evolution Hip Trial database (Clinical trial identifier: NCT01721278) which commenced October 2012(14). Under study ethical approval (Ethics Committee registration (10/H0807/101), consenting subjects had their gait assessed using a treadmill instrumented with force plates. A total of 18 patients were identified as meeting the inclusion criteria of having a long and short stem hip replacement in situ without any other joint or medical disorder to affect gait. However, six subjects were excluded. One subject was excluded due to poor overall balance, which prevented safe assessment. The five other subjects were excluded as they formed a minority who had a variety of other long femoral stems (Modular stem *n* = 1, cemented stem *n* = 2, other uncemented brand = 2), which were different from the rest of the group. An appropriate anthropometrically similar control group without known lower limb disease from the gait lab database was retrieved for analysis, so this meant that a total of 25 subjects with twelve in the arthroplasty and a further thirteen in the control group was analysed. All arthroplasty subjects were implanted with a short stem which is 100 mm from shoulder to tip in length (Furlong Evolution, Joint replacement instruments, Sheffield, England) in one hip and a long stem which is 140–166 mm from shoulder to tip in length (Furlong HAC, Joint replacement instruments, Sheffield, England) in the other hip. Both uncemented stems are hydroxyapatite plasma sprayed with the same mechanical stability principles and ODEP (orthopaedic device evaluation panel) approved, with the Furlong HAC, which has been around longer, having a best 10A* rating. Case records of the arthroplasty group were further screened to confirm an uncomplicated surgical recovery and ensure the absence of other lower limb replacements or conditions, which might have affected walking ability. All subjects were analysed by a blinded assessor to avoid any potential testing bias.

### Radiological assessment

Radiographic pre-operative disease severity was assessed using Kellgren-Lawrence osteoarthritis grading system with orthogonal radiographs of the hip (15). Postoperative radiographs were scrutinised to ensure that an accurate reconstruction of hip off-set, leg length and cup inclination had been achieved (16).

### Surgical intervention and rehabilitation

**Fig. 1:**
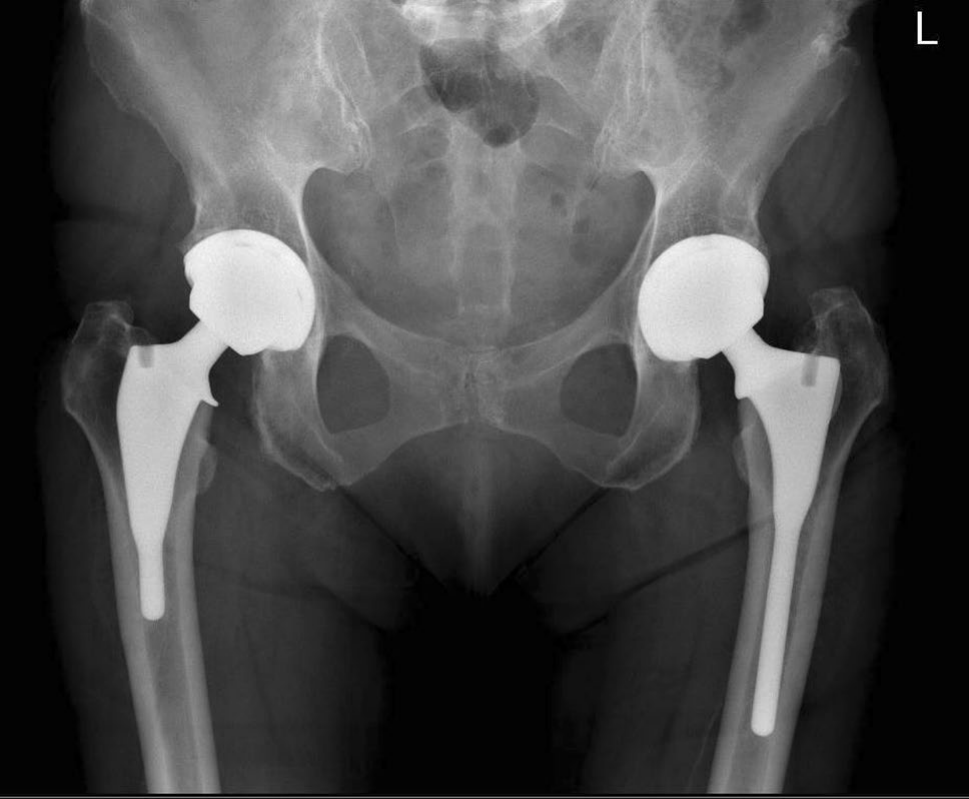
A postoperative weight bearing plain radiograph demonstrating the difference of femoral stem lengths

All surgery was performed by the senior surgical author using a conventional posterior approach with a trans-osseous muscle and capsular repair. The senior surgeons’ implant of choice switched from the long to the short stem following its introduction (Fig. 1). Thus surgery was performed in a staged fashion with long stems being implanted first and short stems second at a later date once contralateral hips were arthritically afflicted and symptomatic. All implanted hips had a ceramic on ceramic bearing couple. All subjects had standard hip precautions and day 1 rehabilitation programme irrespective of the implant used.

### Patient-reported outcome measures (PROMS)

PROMs assessment in the form of the Oxford hip score (OHS) (17), EuroQol 5 part questionnaire (EQ-5D) with the EuroQol visual analogue scale (EQ-VAS) scores (18) were obtained prior gait analysis. The psychometric questionnaires were collected for both hips rather than individually to prevent inducing conscious bias toward a particular side.

### Gait instrumentation, variables, processing and analysis

**Fig. 2:**
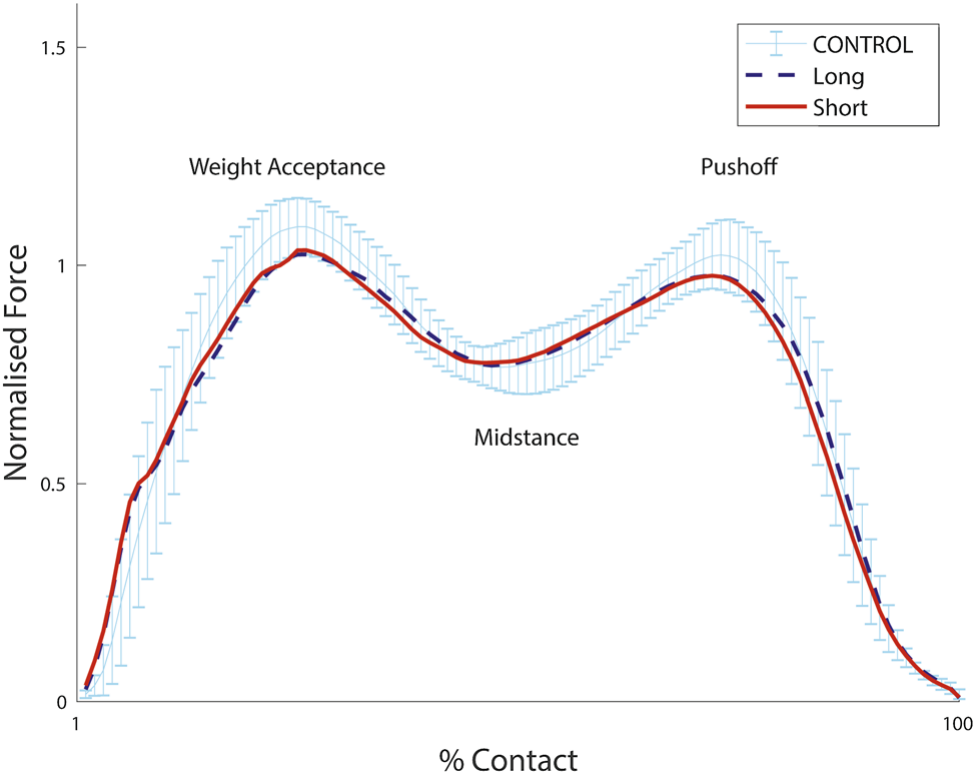
Ground reaction forces during uphill walking (15% uphill at 4 km per hour). The central line is the mean and the whiskers are the 95% confidence interval (CI) of the controls

Gait analysis was conducted on a previously reported protocol using a validated treadmill instrumented with tandem piezo-electric force plates beneath the treadmill belt (Gaitway™ II, Kistler Instrument Corporation, Amherst NY) (12, 13). The vertical component of the ground reaction forces (GRF) were collected by calibrated force plates at a sample frequency of 100 Hz. Ground reaction forces were the focus of analysis as it reflects the load transmitted through the limb and thus will identify any limb predilection or advantage. The chosen variables for analysis, as seen in Fig. 2, were weight acceptance (WA), midstance (MS), and pushoff (PO) based on studies demonstrating excellent reliability and repeatability with an intra-class correlation coefficient of 0.93–0.99 (19, 20). Furthermore, these variables are important for a functionally stable hip as weight acceptance represents the hip in flexion accepting the load transfer of the entire body, while the midstance representing the hip centre of mass going from flexion to extension with it terminating the load transfer by push offing to the opposite limb. All subjects prior to testing were weighed by the treadmill force plate to allow normalisation for body weight as according to Hof et al. (21). All participants had a standardised warmup and acclimatisation period of 6 min to reduce gait data inconsistencies associated with being unfamiliarised (22). Walking pace was incrementally increased from 3 km/h by 0.5 km/h until their top walking speed (TWS) was achieved. TWS was defined as the fastest the subject could comfortably walk without running. The treadmill was then raised to record uphill walking gait data at 5°, 10° and 15° as it is a common everyday activity and shown to influence the condition to test gait (20). GRF data were collected for both limbs at all speed and incline intervals for 10 s each. All trials were visually processed to ensure six consecutive strides were taken cleanly. As a large amount of trials were collected a MATLAB (Mathworks, Mass, USA) script was written to extract the data from the Kistler software in a formatted manner for analysis. Data was stratified into short and long stems for the arthroplasty group and right and left limbs for the healthy control group. A validated symmetry index was completed to assess the gait symmetry of the short stem limb to the contra-lateral opposing limb(23). It was calculated using the formula:

$$\mathrm{Absolute SI}= \frac{\left|X1-X2\right|}{0.5 \times \left(X1+X2\right) } \times 100\%,$$

where X1 was the short stem implanted limb measure and X2 was the contra-lateral conventional limb measure. It gave a measure of percent difference between limbs. X1 and X2 were used for controls right and left respectively.

Statistical analysis was performed with Matlab. All variables were shown to be normally distributed with a Shapiro–Wilk test. All variables for each of the subject group were compared to each other using an analysis of variance (ANOVA) with Tukey post hoc test. For continuous variables between implanted limbs, a paired *T* test was used. A chi-squared test was used for categorical variables. The significance was set at *α* = 0.05 throughout.

A minimum sample size of 9 subjects comprising 18 hips in 2 groups was chosen based on an analogous study design comparing hip resurfacing and conventional THA which demonstrated statistical advantages with a minimum clinical difference of 5% (13). To ensure a difference could be found, a power analysis was conducted with G*Power™ using the above study findings of weight acceptance mean of 1208 and 1279 Newtons force along with a standard deviation of 320 and 390 for the respective arthroplasty hips. With an effect size determinate of 0.59, an alpha error probability of 0.05 and power of 0.80, it determined a total of 20 hips would give an actual power of 0.82 with a noncentrality parameter delta of 2.65 and acritical t of 1.73.

## Results

A total of 25 subjects had analysis. The arthroplasty (*n* = 12) and control (*n* = 13) participants were similarly matched for gender, BMI and height (Table 1) with the controls slightly but not statistically younger. PROMs revealed excellent post-operative patient scores and satisfaction as measured using the OHS and Eq-5D. Due to being the 2nd procedure in all the hip arthroplasty patients, the short stemmed hip replacements had a significantly (*p* < 0.0001) shorter mean follow-up (31vs79 months) from surgery to gait analysis as it is under trial currently with one hip being 10 months postoperatively and the remaining minimum 13 months postoperatively. Both hips of all patients had simple primary osteoarthritis with symmetric pelvic morphology. Both sides had similar preoperative disease severity. In all arthroplasty subjects, the implant positions in terms of hip offset and leg length were within 5 mm of each other (Table 1). Comparable numbers (five long and seven short stems) were implanted in the right hips.

**Table 1:** Subject characteristics, patient reported outcome measures and radiographic hip measurements

Subject	Control Group	THR Group	
Sex M:F	5:8	5:7	
Age (years)	66 (52–85)	73 (59–83)	
BMI	27 (21–35)	26 (19–30)	
Height (cm)	167 (156–180)	169 (158–184)	
Top Speed (km/h)	6.9 (6–7.5)^†^	5.9 (4–7.5)	
Oxford Hip Score	NA	45 (39–48)	
EQ-5D	NA	0.922 (0.735–1.000)	
EQ-VAS	NA	86 (75–100)	
		Short	Long
Pre-op OA Severity	NA	3 (2–4)	3 (2–4)
Follow-up (months)	NA	31 (10–60)^‡^	79 (45–116)
Inserted Side (Right)	NA	7	5
Head Size (mm)	NA	36 (32–40)	36 (32–40)
Cup Inclination (Degrees)	NA	39 (37–43)	41(37–44)
Hip Offset Difference (Short-Long) mm	NA	− 1 (− 5–4)	
Hip Length Difference (Short-Long) mm	NA	1 (-4—5)	

The values are indicated as means (range)

^†^Significant difference between patient groups versus control (*p* < 0.05)

^‡^Significant difference between patient groups (*p* < 0.05)

### Gait analysis

The mean top walking speed for the hip replacement groups was 5.9 km/h which was statistically slower (*p* = 0.006) than the control group who walked 1 km/h faster. In total, 90 gait assessments were analysed for the 12 arthroplasty patients. Predictably, there was a wide range of walking speeds (4–7.5 km/h) and inclines (5–20%) that arthroplasty subjects could achieve.

**Fig. 3:**
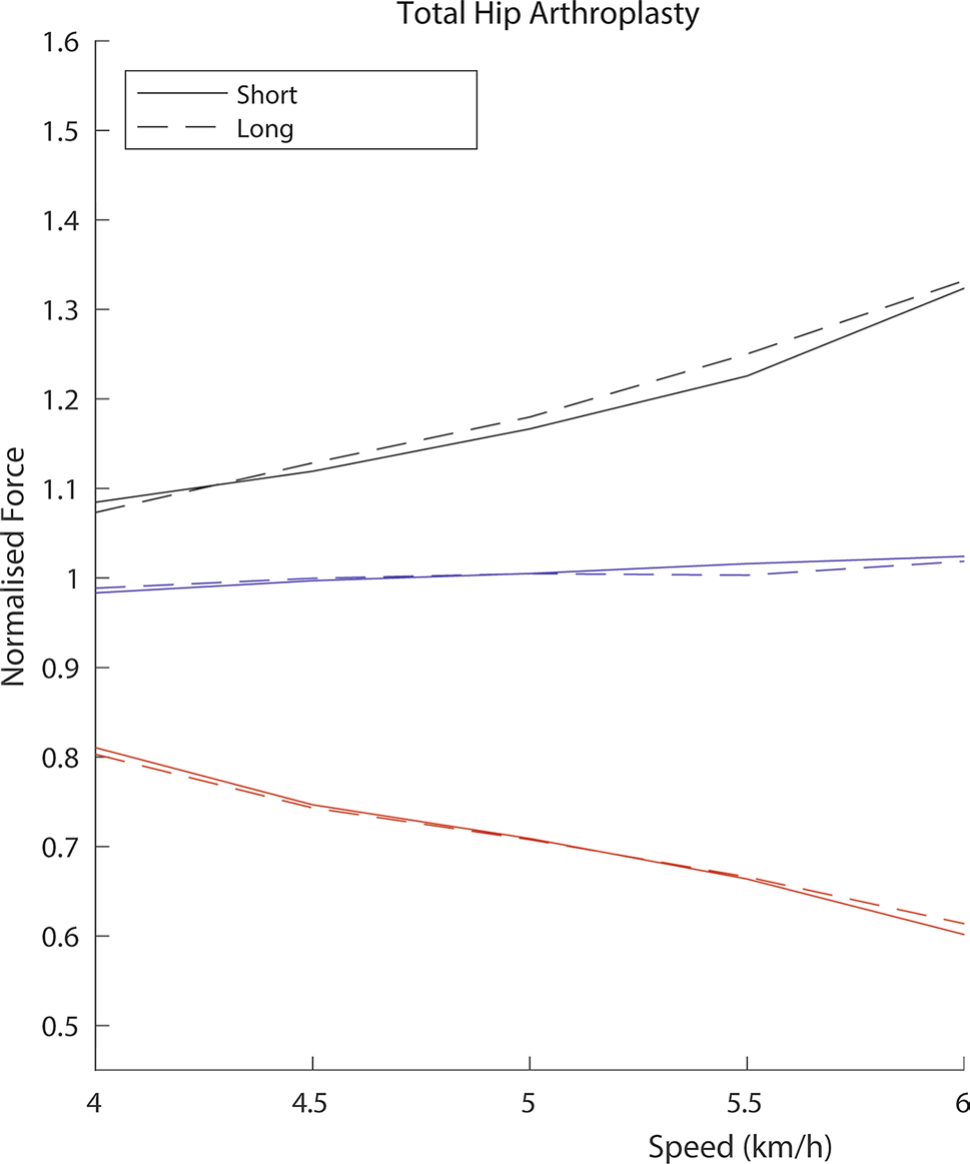
Mean ground reaction force trends at increasing speeds for the arthroplasty group: weight acceptance (top), push-off (middle), and midstance (bottom)

The ground reaction force parameters of long and short stem implanted limbs demonstrated no statistical difference at any specific walking speed or incline assessment (Tables 2 and 3). The visual ground reaction force patterns for short and long stems were also symmetrical (Figs. 2 and 3) demonstrating equivalent weight acceptance, midstance and pushoff. The weight acceptance steadily rose with increasing speeds, but this increase did not transfer to push-off which was lower at higher speeds than the healthy controls (Tables 2 and 3).

**Table 2:** Incline parameters means with mean absolute symmetry indices (SI) in percent %

Variable	Assessment		4 km/h 5% Incline	SI	4 km/h 10% Incline	SI	4 km/h 15%	SI
	Group	Limb						
Weight Acceptance (BWN)	Control	Right	1.07 (0.98–1.19)	2.2	1.07 (0.95–1.20)	3.2	1.11 (1.02–1.16)	1.9
		Left	1.06 (0.90–1.18)		1.06 (0.92–1.22)		1.10 (0.98–1.19)	
	Hip Arthroplasty	Long	1.04 (0.94–1.12)	2.2	1.04 (0.97–1.10)	3.3	1.05 (0.97–1.18)	2.8
		Short	1.05 (0.94–1.15)		1.05 (0.92–1.12)		1.06 (0.99–1.19)	
Midstance (BWN)	Control	Right	0.82 (0.69–0.87)	3.0	0.79 (0.62–0.89)	2.7	0.75 (0.54–0.90)	2.7
		Left	0.82 (0.65–0.86)		0.79 (0.63–0.89)		0.76 (0.56–0.91)	
	Hip Arthroplasty	Long	0.81 (0.69–0.89)	3.0	0.79 (0.71–0.86)	4.5	0.75 (0.67–0.84)	4.5
		Short	0.81 (0.74–0.95)		0.78 (0.70–0.87)		0.77 (0.72–0.86)	
Pushoff (BWN)	Control	Right	1.07 (0.91–1.13)	3.2	1.06 (0.91–1.14)	2.5	1.04 (0.89–1.14)	2.1
		Left	1.05 (0.91–1.17)		1.06 (0.88–1.18)		1.03 (0.91–1.17)	
	Hip Arthroplasty	Long	1.01 (0.91–1.07)	2.1	1.01 (0.88–1.07)	1.7	1.00 (0.95–1.13)	3.0
		Short	1.01 (0.94–1.05)		1.01 (0.88–1.04)		1.00 (0.96–1.09)	

Values are presented as means (range). SI signifies absolute symmetry index in percent %. BWN signifies body weight normalized force

**Table 3:** Speed parameters means with mean absolute symmetry indices (SI) in percent %

Variable	Speed		4 km/h Flat	SI	4.5 km/h Flat	SI	5 km/h Flat	SI	5.5 km/h Flat	SI	6 km/h Flat	SI
	Groups	Limb										
Weight Acceptance (BWN)	Control	Right	1.09 (0.99–1.16)	3.0	1.12 (1.06–1.18)	3.4	1.17 (1.09–1.24)	3.3	1.23 (1.14–1.35)	2.4	1.30 (1.21–1.40)	2.9
		Left	1.08 (0.97–1.19)		1.10 (1.00–1.22)		1.16 (1.05–1.29)		1.23 (1.17–1.38)		1.29 (1.20–1.42)	
	Hip Arthroplasty	Long	1.07 (0.99–1.18)	2.7	1.13 (1.01–1.28)	2.6	1.18 (1.11–1.30)	3.1	1.25 (1.14–1.46)	4.2	1.33 (1.20–1.50)	5.7
		Short	1.08 (1.01–1.18)		1.12 (1.03–1.27)		1.17 (1.10–1.31)		1.23 (1.08–1.42)		1.32 (1.22–1.58)	
Midstance (BWN)	Control	Right	0.81 (0.74–0.84)	3.3	0.78 (0.67–0.87)	4.2	0.74 (0.61–0.83)	3.3	0.69 (0.56–0.78)	3.2	0.63 (0.53–0.74)	4.7
		Left	0.83 (0.72–0.86)		0.80 (0.73–0.88)		0.75 (0.65–0.86)		0.69 (0.58–0.79)		0.64 (0.55–0.71)	
	Hip Arthroplasty	Long	0.80 (0.69–0.89)	1.7	0.74 (0.65–0.81)	2.4	0.71 (0.65–0.80)	2.6	0.67 (0.59–0.78)	3.3	0.61 (0.50–0.75)	4.6
		Short	0.81 (0.74–0.95)		0.75 (0.67–0.81)		0.71 (0.63–0.78)		0.66 (0.57–0.74)		0.60 (0.51–0.72)	
Pushoff (BWN)	Control	Right	1.03 (0.93–1.18)	2.5	1.04 (0.93–1.15)	2.3	1.07 (0.96–1.21)	2.6	1.07 (0.96–1.18)	3.9	1.06 (0.92–1.14)	3.1
		Left	1.02 (0.90–1.17)		1.03 (0.92–1.14)		1.05 (0.90–1.20)		1.05 (0.93–1.17)		1.06 (0.92–1.17)	
	Hip Arthroplasty	Long	0.99 (0.91–1.13)	2.4	1.00 (0.83–1.12)	2.7	1.01 (0.83–1.12)	2.4	1.00 (0.79–1.13)	3.7	1.02 (0.80–1.11)	3.2
		Short	0.98 (0.92–1.08)		1.00 (0.81–1.09)		1.01 (0.82–1.13)		1.01 (0.79–1.19)		1.02 (0.76–1.14)	

Values are presented as means (range). SI signifies absolute symmetry index in percent %. BWN signifies body weight normalized force

When comparing the ground reaction force parameters between the implants at all walking speeds, and inclines, both had a near equal distribution of preference for side, with 51% favouring the short and 49% favouring the long stem side. During weight acceptance, the preferential side was marginally greater with 56% favouring the short and only 44% favouring the long side. While during pushoff, the long was the favoured side 53% of the time and the short 47% of the time. During midstance, as found in total, 51% of the time the short was the favoured side compared to 49% for the long stem. As a whole, there was no statistical difference with near-identical mean body weight normalised weight acceptance (1.18 vs 1.18), midstance (0.71 vs 0.71), pushoff (1.00 vs 1.01) during all level walking speeds for long and short stems, respectively. This was also found during all incline walking analysis with a mean body weight normalised weight acceptance (1.04 vs 1.04), midstance (0.78 vs 0.78), and pushoff (1.01 vs1.01) for long and short stem implants respectively.

## Discussion

This unique study set out to determine the impact of reducing the femoral stem length on gait by assessing patients with both a long (*n* = 12) and short (*n* = 12) THA type on contralateral sides while walking at a variety of speeds and inclines. The most important finding of this study is that the short-stemmed device demonstrated functional equivalence in all phases of loading during stance when compared to a reputable long stem with greater than 95% survival beyond 10 years (24, 25). While not demonstrating any performance advantage, it simultaneously and clearly demonstrated no disadvantage when compared to a device which requires 30–40% (100 mmvs140–166 mm) more anatomical disruption to get implantation. These findings are consistent with an earlier gait study with the same implants in differing patient groups which demonstrated similar loading features during weight acceptance, midstance and pushoff (12). These observations support the growing evidence that a shorter stemmed device used for total hip arthroplasty performs well when considering an everyday activity, such as walking, which up to this point has not been fully understood (5, 12). Though both hip implants worked well in comparison for symmetry, there were noted trends of poorer ground reaction forces in each hip when compared to healthy controls but this did not reach statistical difference. The greatest difference was noted during uphill walking and faster walking with as much as 6% variance which is consistent with the previous study showing statistical differences during this phase in terminal hip extension (12). The conclusion that stem length has no impact on function may represent a type II statistical error, a methodological limitation of this small group of subjects. Whilst not statistically significant in this study, the small differences found might prove to be clinically relevant and further research should concentrate particularly on the weakness of pushoff postoperatively. The reasons for gait differences between controls and arthroplasty subjects are likely to be multifactorial. Ageing subjects with degenerative disease who have had multiple surgical procedures may simply be the cause of this weakness; however surgical approach and implant geometry may also play a part which may be of interest for future studies.

The fundamental limitation of the study is the retrospective nature which does not observe the change over time with differing stem types. Furthermore, the postoperative period to gait analysis was shorter in the side of the short stem, including one case with a follow-up of 10 months. Despite that the strict inclusion/exclusion selection process meant that the groups were unique and uniform in terms of approach, fixation method, and implant position. This allowed the study to remove any patient associated confounding variables and focus on the loading characteristics of differing stem lengths in individuals during a period of rejuvenescence considering the overall follow-up period. This was supported by a prized prospective study which found no further significant functional improvement after 6 months when compared to 12 months (26). Finally, the last limitation is that of limb dominance and laterality as assymetries may exist (27), fortuitously in our hip arthroplasty group there were similar numbers implanted on the right side with seven for the short and five for the conventional stems respectively.

The strengths of the current study include the use of an age, gender and BMI similar control group which allowed a worthy comparison of an ordinary activity of daily living expected following surgery. Furthermore, the extensive and robust gait assessment protocol gave the reader a wide range of gait trends, providing an improved understanding of the functional limits that can be achieved by reducing the stem length in THA. The method of between limb evaluation in the same patient ensures for a fairer test which removes patient selection bias which up till now has been a criticism of previous cohort studies.

In conclusion, this study has revealed that reducing the femoral stem length has no performance impairment on gait, whilst demonstrating functional equivalence when compared to a highly rated long-stemmed THA. Its use in vivo nearly 3 years on average following implantation looks promising, and its ongoing assessment as part of a multicentre study should provide important data as to its efficacy longer- term.
